# A Peptide against Soluble Guanylyl Cyclase α1: A New Approach to Treating Prostate Cancer

**DOI:** 10.1371/journal.pone.0064189

**Published:** 2013-05-27

**Authors:** Shuai Gao, Chen-Lin Hsieh, Meenakshi Bhansali, Archana Kannan, Lirim Shemshedini

**Affiliations:** Department of Biological Sciences, University of Toledo, Toledo, Ohio, United States of America; Innsbruck Medical University, Austria

## Abstract

Among the many identified androgen-regulated genes, sGCα1 (soluble guanylyl cyclase α1) appears to play a pivotal role in mediating the pro-cancer effects of androgens and androgen receptor. The classical role for sGCα1 is to heterodimerize with the sGCβ1 subunit, forming sGC, the enzyme that mediates nitric oxide signaling by catalyzing the synthesis of cyclic guanosine monophosphate. Our published data show that sGCα1 can drive prostate cancer cell proliferation independent of hormone and provide cancer cells a pro-survival function, via a novel mechanism for p53 inhibition, both of which are independent of sGCβ1, NO, and cGMP. All of these properties make sGCα1 an important novel target for prostate cancer therapy. Thus, peptides were designed targeting sGCα1 with the aim of disrupting this protein’s pro-cancer activities. One peptide (A-8R) was determined to be strongly cytotoxic to prostate cancer cells, rapidly inducing apoptosis. Cytotoxicity was observed in both hormone-dependent and, significantly, hormone-refractory prostate cancer cells, opening the possibility that this peptide can be used to treat the usually lethal castration-resistant prostate cancer. In mouse xenograft studies, Peptide A-8R was able to stop tumor growth of not only hormone-dependent cells, but most importantly from hormone-independent cells. In addition, the mechanism of Peptide A cytotoxicity is generation of reactive oxygen species, which recently have been recognized as a major mode of action of important cancer drugs. Thus, this paper provides strong evidence that targeting an important AR-regulated gene is a new paradigm for effective prostate cancer therapy.

## Introduction

One important target tissue of androgens and androgen receptor (AR) is the prostate. Like the development of normal prostate, the growth and progression of prostate cancer are also dependent on androgens and AR [1]. In both normal prostate development and prostate carcinogenesis, androgens and the AR are important in regulating the proliferation and survival of prostate cells [2]. Androgen ablation by castration in rats leads to decreased proliferation and increased apoptosis of prostate luminal epithelial cells, resulting in the regression of the prostate gland. When physiological levels of androgens are replaced in a castrated rat, prostate epithelial cell proliferation is increased and apoptosis is decreased, leading to reconstitution of a normal prostate [3]. Recently, it was shown that mutation of the AR is sufficient for causing prostate cancer development and progression [4] and that overexpression of AR converts prostate cancer growth from androgen-dependent to androgen-independent [5]. All the data accumulated thus far strongly suggest that androgens, through the activity of AR, regulate the rate of cellular proliferation while inhibiting the rate of cell death in the prostate [6]. Dysregulation of this balance between cell proliferation and cell death is undoubtedly critical to the development of prostate cancer.

We have previously shown that one important mediator of prostate cancer cell proliferation is soluble guanylyl cyclase α1 (sGCα1; gene name *GUCY1A3*) [7]. sGCα1 was originally identified as a component of sGC, a heterodimeric enzyme, consisting of sGCα1 and sGCβ1 subunits, that mediates biological functions of nitric oxide (NO) [8]. In this physiologically important and ubiquitous signaling pathway, NO binds to and activates sGC, leading to the formation of the secondary messenger cGMP (3′, 5′-cyclic guanosine monophosphate), which then activates a variety of downstream targets, including protein kinase G [9]. Our lab recently identified sGCα1 as a novel AR-regulated gene [7]. We have shown that the sGCα1 promoter is a target of AR regulation and results in greatly higher protein levels of sGCα1 than sGCβ1 in LNCaP cells [7]. sGCα1 is essential for the growth of both androgen-dependent and androgen–independent prostate cancer cells. Importantly, this effect is independent of sGCβ1, NO, and cGMP, and thus sGC enzyme activity [7]. In addition, sGCα1 expression is barely detectable in normal prostate tissues and is markedly elevated in prostate cancer tissues, with expression levels increasing with increasing stage of disease and the highest levels observed in hormone-refractory prostate cancer [7]. Most recently, our lab reported that sGCα1 can block the activity of p53 in and thus enhance the survival of prostate cancer cells [10].

All those pro-cancer functions of sGCα1 suggest that this protein may be a good target for prostate cancer therapy. To address this, we used our previous data showing that sGCβ1 and sGC enzyme activity were not involved in sGCα1 pro-cancer functions and thus hypothesized that sGCβ1 dimerization with sGCα1 can disrupt its pro-proliferation and pro-survival functions. This led us to consider a peptide-based approach for disrupting sGCα1 pro-cancer functions, designing peptides that mimicked the sGCβ1 heterodimerization domains. We hypothesized that such peptides would be able to bind specifically to sGCα1 and disrupt its functions. Among the four peptides used, two of them exhibited cytotoxic activity against prostate cancer cells. One peptide, called A-8R, showed the strongest activity and thus was selected for further study. Peptide A-8R was not only cytotoxic to cultured cells, but also had strong anti-cancer activity against castration-resistant prostate tumors in mouse xenograft studies. Furthermore, our data show that Peptide A-8R kills cancer cells by generation of reactive oxygen species (ROS) and induction of DNA damage. Our findings here identify a new peptide that can arrest the growth of castration-resistant prostate cancer (CRPC).

## Materials and Methods

### Cell Culture and siRNA Transfection

LNCaP, C81, PC-3 and Cos cells were grown as previously described [7]. CWR-22Rv1 cells were cultured in RMPI-1640 medium with 10% FBS and 50 µg/ml Gentamicin (Gibco). Control siRNA, sGCα1 siRNA and AKT siRNA (Dharmacon) at 50 nM final concentration was transfected into cells using Lipofectamine siMAX (Invitrogen).

### Generation of Stable Cell Lines

Generation of stable cell lines was described previously [11]. LNCaP cells were transfected with 2 µg each of pCI-Neo vector (negative control from Promega), sGCα1/pCI-Neo using Lipofectamine 2000 (Invitrogen), and selected in RPMI 1640 complete medium containing 0.9 mg/ml neomycin (Sigma). The colonies were selected by detecting sGCα1 mRNA and protein levels using PCR and Western blotting.

### Peptide Synthesis

All peptides used in this study were synthesized by ChiScientific, at ≥95% purity, and were dissolved in 70% DMSO (ACROS Organic).

### Adenovirus Infection

C81 cells were infected with 5–50 MOI of either an adenovirus expressing Akt (SignaGen) or an empty adenovirus as a control. After 48 hours, the cells were subjected to peptide treatment, followed by Western blotting or proliferation assay.

### Proliferation and Apoptosis Assays

For proliferation, cells were grown in medium containing 2% FBS extracted with dextran-coated charcoal (DCC). 48 hours later, ethanol or 1 nM R1881 was added to the cells followed by peptide treatment. The MTT assay (Sigma) was used as before [11] to determine cell number. For apoptosis, 5000 cells were seeded in 96-well plates and treated with Vehicle (DMSO), Peptide A-8R (25 µM), Etoposide (50 µM) (Sigma) at different time points. The Caspase (3/7) activity was measured using Apo-ONE Homogeneous Caspase-3/7 assay kit (Promega). PARP cleavage was also used to measure apoptosis, and is described below.

### Western Blotting

Western blotting was performed as described [7] using primary antibodies against sGCα1 (Cayman Chemical), pan-Akt (Cell Signaling Technology), phospho-AKT (S473, T308) (Cell Signaling Technology), phospho-PDK1 (Cell Signaling Technology), phospho-GSK-3β (Cell Signaling Technology), PARP (Cell Signaling Technology), and β-Actin (Abcam).

### Immunocytochemistry

Immunocytochemistry was used to study the subcellular localization of sGCα1 and Biotin-labeled Peptide-8R in LNCaP cells. FITC-labeled anti-sGCα1 antibody (1∶100 dilution; Santa Cruz Biotechnology), anti-Biotin antibody (1∶200; Santa Cruz Biotechnology) were used for immunocytochemisty as described [12].

### Immunoprecipitation, Biotin Pulldown, and Binding Affinity

Immunoprecipitation (IP) in LNCaP cells was performed as described previously [10]. Whole-cell extracts from LNCaP cells were subjected to IP using Protein A/G plus Agarose (Santa Cruz). IP antibodies were against sGCα1 (Cayman Chemical), sGCβ1 (Cayman Chemical), or rabbit IgG (Santa Cruz) as control. For Biotin pulldown, 5 µg Biotin-labeled Peptide A was incubated with NeutrAvidin Agarose Resin (Thermo Scientific) for 3 hours at 4°C, after which whole-cell extract from LNCaP cells was added and incubated overnight at 4°C. Resin was washed and eluted with SDS Sample buffer, followed by SDS-PAGE gel electrophoresis. For the competition assay, the same experiment was repeated with the following change: LNCaP cell extract was divided into 3 equal parts, with each part receiving 5 µg Peptide A-8R-Biotin and Vehicle, 150 µg Peptide C-8R, or 150 µg Peptide A-8R.

For binding affinity experiments, whole-cell extract from C81 cells was incubated with different concentrations of Peptide A-8R tagged with FITC at room temperature for 2 hours. IPs were performed using antibodies against sGCα1 or rabbit IgG as control. Peptide A-8R-FITC pulldown was measured by fluorescence signal at excitation and emission wavelengths of 485 and 521 nm, respectively, using a multi-well fluorescence plate reader.

### ROS Measurement and Rescue

Intracellular ROS levels were evaluated using the fluorescent probe 5-(and-6)-chloromethyl-2′,7′-dichlorodihydrofluorescein diacetate (CM-H_2_DCFDA) (Invitrogen). After treatments with Peptide A-8R, C81 cells were loaded with 20 µM CM-H_2_DCFDA and incubated at 37°C for 30 min in the dark. Cells were then washed with PBS and fluorescence was measured at the excitation and emission wavelengths of 490 and 535 nm, respectively, using a multi-well fluorescence plate reader. For NAC (N-acetyl cysteine) (Sigma) rescue experiment, cells were pretreated with 1 or 5 mM NAC for 2 hours before adding Peptide A-8R.

### Comet Assay

C81 cells were treated with Peptide A-8R for 1 hour, following a 2-hour pretreatment with 5 mM NAC or Vehicle, and trypsinized, and mixed with Comet LM-Agarose (CometAssay, TREVIGEN) and transferred to slides. Electrophoresis and Sybr-Green staining was carried out according to manufacturer’s protocol.

### Mouse Xenograft Tumor Studies

2×10^6^ C81 cells in 50 µL of growth medium was mixed with 50 µL Matrigel (BD Biosciences) and injected into both flanks of 4–6-week old SCID male mice. When tumor sizes reached 200 mm^3^, 50 µL of Peptide A-8R (40 mg Peptide A-8R/Kg of animal) or Vehicle (DMSO) was directly injected into the tumors every other day. Injections were stopped after 5 times and tumors were measured every 3 days. Mice were euthanized after 3 weeks and tumors were excised. All procedures were approved by the University of Toledo Division of Lab Animal Recourses (DLAR). Protein extracts were prepared by boiling the tumor tissue in 3× SDS buffer, and evaluated by SDS-PAGE gel.

This study was carried out in strict accordance with the recommendations in the Guide for the Care and Use of Laboratory Animals of the National Institutes of Health. The protocol was approved by the Institutional Animal Care and Use Committee of the University of Toledo (Protocol Number: 106418). During the experiments all animals were monitored daily for morbidity and all efforts were made to minimize suffering. At the end of the study all animals were euthanized using inhalation of carbon dioxide, after which surgery was performed to excise tumors from all animals.

## Results

### Peptides Targeting sGCα1 are Cytotoxic to Prostate Cancer Cells

**Figure 1:**
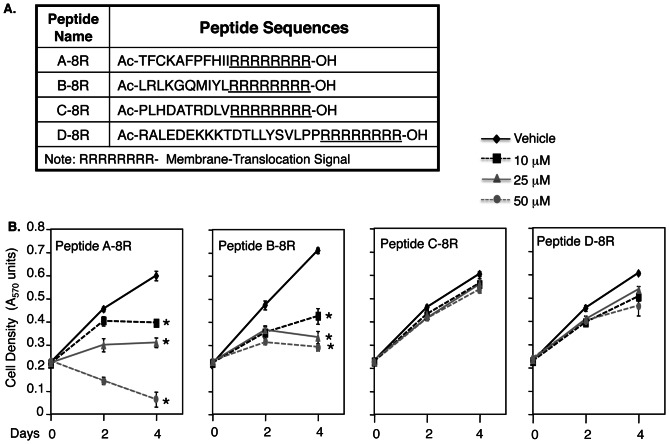
Among four peptides, Peptide A-8R is strongly cytotoxic to prostate cancer cells. (**A**) Four peptides were designed targeting sGCα1, with each peptide containing eight arginines at the C-terminus for membrane translocation. (**B**) LNCaP cells were grown in 10% serum under different concentrations of the four peptides, as shown. Cell number was measured after 0–4 days of incubation. Data points represent averages of three independent experiments plus standard deviations. Asterisks indicate statistical significance (P<0.0002) of Peptide A activity, relative to Vehicle.

Our data demonstrating that sGCα1 is required for the survival [10] and proliferation [7] of prostate cancer cells suggest that this protein may be a good target for prostate cancer therapy. To begin to address this possibility, we used these previous data showing that sGCα1 pro-cancer functions are independent of NO signaling and sGCβ1 [7], [10] and that sGCβ1 can relieve sGCα1-mediated repression of p53 transcriptional activity [10], suggesting that sGCβ1 dimerization with sGCα1 can disrupt sGCα1 pro-cancer functions. This led us to consider a peptide-based approach for disrupting the sGCα1 functions, using peptides that mimic the four known sGCβ1 heterodimerization domains [13]. These peptides, named Peptide A, B, C, and D, varied in length from 11 to 19 amino acids. Each peptide contained 8 arginines at the carboxy terminus (Fig. 1A), a sequence known to mediate plasma membrane translocation and cellular internalization [14]. The peptides were tested for activity on LNCaP cells, a cell line that expresses both AR and sGCα1 [7]. As shown in Fig. 1B, Peptide A-8R had a strong, dose-dependent, cytotoxic activity, killing nearly all cells by day 4. Peptide B-8R also had a negative effect, suppressing cell growth but not reducing cell number as Peptide A-8R did (Fig. 1B). On the other hand, Peptides C-8R and D-8R had little effect (Fig. 1B). In view of its strong cytotoxic activity, Peptide A-8R was selected for further study.

### Peptide a Associates with Endogenous sGCα1 in Prostate Cancer Cells

**Figure 2:**
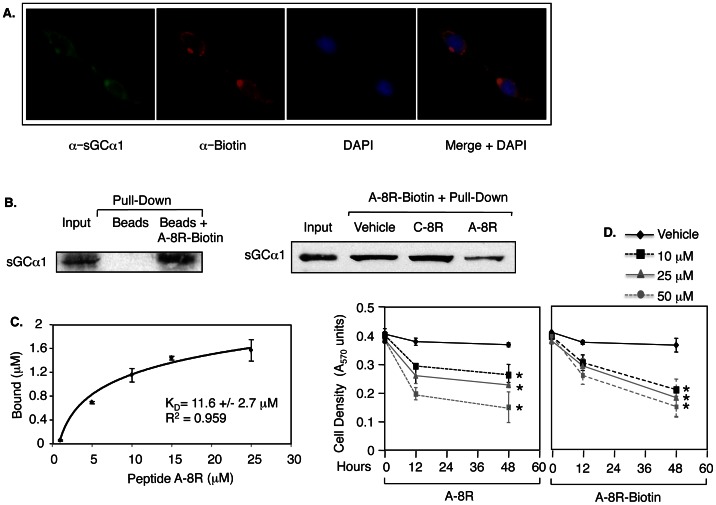
Peptide A-8R interacts with sGCα1 in prostate cancer cells. (**A**) LNCaP cells were treated with 25 µM Biotin-labeled Peptide A-8R for 2 hrs and subjected to immunocytochemistry using anti-sGCα1 or anti-Biotin antibody to measure subcellular co-localization of endogenous sGCα1 and Peptide A-8R. DAPI was used to stain nuclei. (**B**) LNCaP cytosolic extracts were incubated with Peptide A-8R-Biotin and subjected to purification using streptavidin-agarose. This pull-down experiment was repeated with competing excess amount of unlabeled Peptide C-8R or Peptide A-8R. In both cases Western blotting was used to measure the co-purification of sGCα1. (**C**) LNCaP cell extract was incubated with different concentrations of Peptide A-8R-FITC and endogenous sGCα1 was immunoprecipitated. Co-purified Peptide A-8R-FITC was quantified by measuring fluorescence signal emission. Nonlinear regression analysis was used to determine an affinity for Peptide A-8R binding to sGCα1. (**D**) LNCaP cells were grown in 10% serum under different concentrations of Peptide A-8R or A-8R-Biotin, as shown. Cell number was measured after 0–48 hrs of incubation. Data points represent averages of three independent experiments plus standard deviations. Asterisks indicate statistical significance (P<0.02) of Peptide A activity, relative to Vehicle.

Peptide A-8R was designed to interact with sGCα1, which has been confirmed using three methods. Peptide A-8R was synthesized with a Biotin tag at the C-terminus, giving Peptide A-8R-Biotin. In the first assay, LNCaP cells were treated with Peptide A-8R-Biotin and subjected to immunocytochemistry using an antibody against Biotin to detect the tagged Peptide A-8R and another against sGCα1 to detect this protein (Fig. 2A). As observed previously, endogenous sGCα1 was found exclusively in the cytoplasm of LNCaP cells, surrounding the nucleus [7]. Importantly, Peptide A-8R-Biotin was also found in the cytoplasm and colocalizes with sGCα1, as shown by the merged images. These results suggest that Peptide A-8R-Biotin interacts with endogenous sGCα1, which was verified by a pull-down experiment. In this second assay (Fig. 2B), LNCaP whole-cell extract was incubated with Peptide A-8R-Biotin and subjected to StreptAvidin-agarose purification, leading to co-purification of sGCα1. In contrast, the agarose beads were unable to pull-down sGCα1 in the absence of Peptide A-8R-Biotin. To measure the specificity of binding, the pull-down experiment was repeated under conditions in which excess amount of unlabeled Peptide A-8R or Peptide C-8R was added to the reaction with Biotin-labeled Peptide A-8R. As shown in Fig. 2B, Peptide A-8R significantly inhibited Peptide A-8R-Biotin interaction with sGCα1, while Peptide C-8R had no effect, showing a specific interaction of Peptide A-8R with sGCα1. We utilized immunoprecipitation (IP) of sGCα1 to determine its binding affinity for Peptide A-8R. Peptide A-8R labeled with FITC was used to determine that the peptide binding affinity (K_D_) for sGCα1 was 11.6 µM (Fig. 2C), which falls within the active range of concentration for Peptide A-8R-mediated cytotoxicity. Collectively, these results confirm a specific physical association between Peptide A-8R and sGCα1. Fig. 2D shows that addition of a Biotin tag to Peptide A-8R did not affect its cytotoxic efficacy.

### Peptide A does not Affect sGC Enzyme Activity

Since Peptide A was designed to mimic an sGCβ1 heterodimerization domain, it is possible that this peptide may affect sGCα1-sGCβ1 heterodimerization and thus enzyme activity. To address the first possibility, we directly measured the interaction of sGCα1 with sGCβ1 by IP using antibodies against sGCα1 and sGCβ1. Peptide A-8R had no effect on either sGCα1 co-IP with sGCβ1 or sGCβ1 co-IP with sGCα1 (Fig. S1A), clearly demonstrating that Peptide A does not disrupt sGCα1-sGCβ1 heterodimerization. Confirmation for this was obtained from an ELISA experiment measuring cGMP synthesis. As shown in Fig. S1B, R1881 treatment markedly elevated cGMP levels, consistent with our previously published data showing androgen induction of sGCα1 expression and cGMP synthesis [7]. Importantly, Peptide A-8R was unable to significantly repress cGMP levels, in either the absence or presence of androgen (Fig. S1B). These data collectively demonstrate that Peptide A does not disrupt sGC enzyme activity and thus suggest that NO signaling is not involved in its cytotoxicity. To directly test this, we added 8-Br-cGMP to treated cells. 8-Br-cGMP had no effect on Vehicle- or Peptide A-8R-treated cells, while it was able to partially rescue cells treated with the sGC enzyme inhibitor ODQ (Fig. S1C). A second approach was to add the NO sequestering agent C-PTIO, which enhanced rather than relieved the cytotoxicity of Peptide A-8R at 10 and 25 µM, but interestingly not 50 µM (Fig. S1D). These data collectively show that Peptide A-8R does not interfere with nor depend on NO signaling.

### Peptide A Blocks the Growth of Both Hormone-dependent and Castration-resistant Prostate Cancer Cells, but not sGCα1-deficient Cells

**Figure 3:**
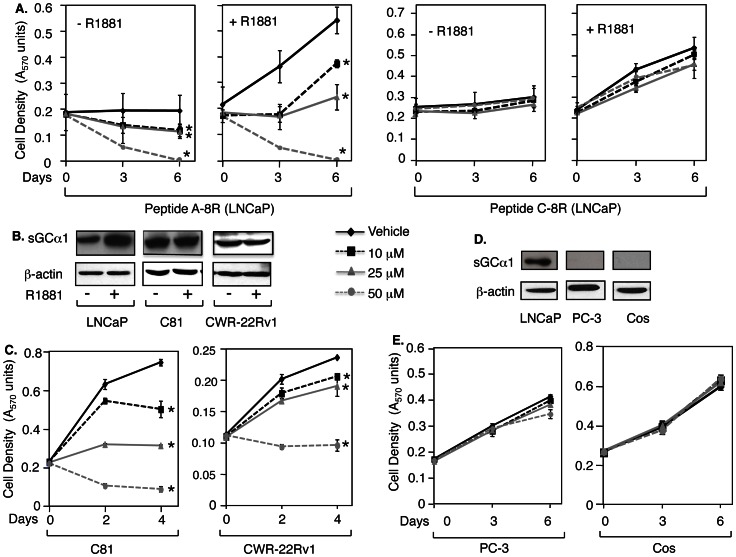
Peptide A-8R is strongly cytotoxic to sGCα1-expressing prostate cancer cells, but not to sGCα1-deficient cells. Vehicle or different concentrations of Peptide A-8R or C-8R, as shown, was added to (**A**) hormone-dependent LNCaP cells grown in 2% serum without or with 1 nM R1881, (**C**) castration-resistant C81 or CWR-22Rv1 cells, or (**E**) sGCα1-deficient PC-3 or Cos cells. Cell number was measured after various days of incubation. Data points represent averages of three independent experiments plus standard deviations. Asterisks indicate statistical significance (P<0.03) of Peptide A activity, relative to Vehicle. Western blotting was used to monitor expression of endogenous sGCα1 in (**B**) C81 and CWR-22Rv1 cells, treated without or with 1 nM R1881, or (**E**) PC-3 and Cos cells, as compared to LNCaP cells. Note that β-actin was used to control for protein loading.

To determine if androgen influenced the cytotoxic activity of Peptide A-8R, the experiment in Fig. 1B was repeated in the presence of hormone. As Fig. 3A shows, Peptide A-8R had the same potent cytotoxic activity with or without androgen, causing all cells to perish by day 6 when treated with 50 µM peptide. An inactive peptide, Peptide C-8R, had no significant effect at the same concentration (Fig. 3A), demonstrating that the amino-acid sequence of Peptide A was required for the cytotoxic effect and excluding potential cytotoxicity induced by the 8-arginine sequence.

To investigate whether the membrane translocation signal is required for the cytotoxicity of Peptide A-8R, we analyzed the activity of Peptide A lacking 8 arginines. As shown in Fig. S2, Peptide A without the arginines had little negative effect, in contrast to the strong cytotoxic effect of Peptide A-8R. These data strongly suggest that cellular internalization is required for the peptide cytotoxic effect.

We previously have shown that sGCα1 promotes prostate cancer proliferation, and its expression increases in higher stage of prostate cancer [7]. As shown previously, sGCα1 protein expression is up-regulated by androgen in androgen-dependent cells, and constitutive in androgen-independent C81 and CWR-22Rv1 cells, with CWR-22Rv1 cells expressing lower levels than C81 cells (Fig. 3B). Thus, we were interested to study the peptide effect on these hormone-refractory prostate cancer cells. Peptide A-8R was very effective at suppressing the growth of C81 cells (Fig. 3C), matching the effect on hormone-dependent LNCaP cells. Peptide A-8R was also effective on another hormone-refractory prostate cancer cell line, CWR-22Rv1 cells, which are distinct from LNCaP cells (Fig. 3C). Importantly, these data demonstrate that hormone-refractory prostate cancer cells are sensitive to the cytotoxic effect of Peptide A-8R as are hormone-dependent cells, suggesting that this peptide may be effective against CRPC, the lethal form of the disease.

Endogenous sGCα1 is expressed in LNCaP, C81, and CWR-22Rv1 cells (see Fig. 3B), all of which are sensitive to the cytotoxic effect of Peptide A-8R. These data suggest that the peptide effect requires endogenous sGCα1 expression, an expected finding since sGCα1 was the designed target of Peptide A-8R. To obtain more evidence for this hypothesis, two cancer cell lines were studied that do not express endogenous sGCα1 (Fig. 3D). Peptide A-8R had little to no effect on PC-3 (prostate cancer) or Cos (mouse kidney cancer) cells (Fig. 3E). These data strongly support the contention that the cytotoxic activity of Peptide A-8R depends on endogenous sGCα1 protein.

### Peptide A Down-regulates AKT in Prostate Cancer Cells

To understand how sGCα1 is mediating cell proliferation, we used an inhibitor of either MAPK or PI3K-AKT signaling, two important signaling pathways regulating cell survival and proliferation. Interestingly, the PI3K-AKT inhibitor LY294002 dramatically repressed LNCaP cell proliferation (Fig. S3A). This led us to explore the possibility that sGCα1 may induce proliferation via this signaling pathway. Using stable LNCaP cell lines over-expressing sGCα1 (Fig. S3B), which exhibit enhanced androgen-induced proliferation (Fig. S3C), we found by Western blotting that levels of total AKT and phosphorylated AKT are greatly elevated in these LNα1-6 and LNα1-4 cells (Fig. S3B); these cells also expressed higher levels of phosphorylated PDK1, a kinase acting on AKT, and GSK-3β, a target of AKT [15]. To confirm that the increased AKT levels were due to sGCα1 over-expression, siRNA was used to knockdown expression of sGCα1 in LNα1-6 cells, resulting in significantly reduced levels of total and phosphorylated AKT (Fig. S3D). Importantly, sGCα1 did not influence the levels of AKT mRNA (data not shown), suggesting that sGCα1 acts on the AKT protein, perhaps affecting its stability. Interestingly, siRNA knockdown of sGCα1 (Fig. S3F) greatly inhibited the growth of LNCaP cells (Fig. S3E), clearly demonstrating that endogenous sGCα1 provides the cells a survival and pro-growth function.

In view of our data above (see Figs. S3B, S3D) suggesting that sGCα1 acts on the AKT protein, Peptide A-8R binding to sGCα1 might be expected to affect AKT. Indeed, treatment of LNCaP cells with Peptide A-8R had a strong, time-dependent negative effect on the AKT protein, such that by 50 min most of the measurable AKT is gone (Fig. S4A). As would be expected, phosphorylated AKT was similarly affected (Fig. S4A). The inactive control Peptide C-8R had no effect on either total AKT or phosphorylated AKT levels (Fig. S4A). Interestingly, the same negative effect on AKT protein levels (Fig. S4B) was observed in mouse xenograft tumors (see below) treated with Peptide A-8R, as observed in LNCaP cells (Fig. S4A).

These results are consistent with the hypothesis that Peptide A-8R disrupts the pro-cancer functions of sGCα1 and suggest that at least one mechanism of its cytotoxic action is via disruption of the AKT protein. To directly test this hypothesis, we over-expressed AKT using an adenovirus system, which resulted in significantly elevated levels of AKT in cells treated with 10 and 25 µM of Peptide A-8R (Fig. S4C). Surprisingly, adenovirus-expressed AKT was unable to rescue the cells treated with 10- 50 µM Peptide A-8R (Fig. S4D), suggesting that AKT down-regulation is not involved in Peptide A-8R-mediated cytotoxicity.

### Peptide A Kills Prostate Cancer Cells by Generation of ROS

**Figure 4:**
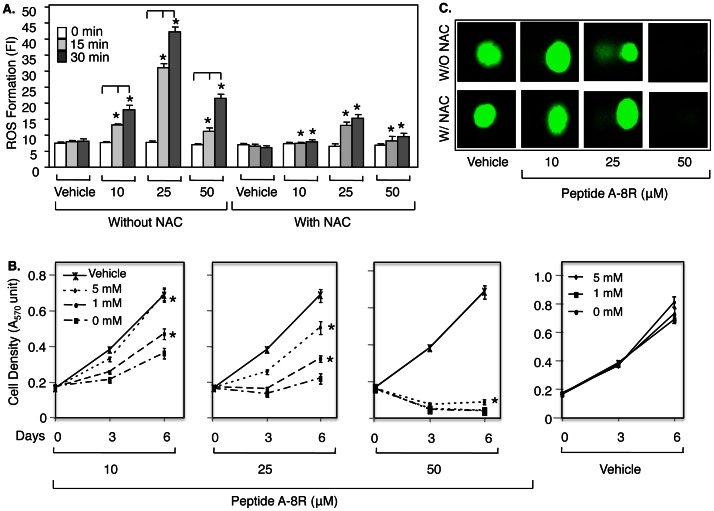
Peptide A-8R kills prostate cancer cells by mediating ROS generation. (**A**) C81 cells were treated with Vehicle or different µMolar concentrations of Peptide A-8R and without or with 0–5 mM NAC and monitored for ROS generation after 0–30 min as measured by fluorescence intensity (FI). Bar graphs represent averages of three independent experiments plus standard deviations. Note that asterisks on bar graphs found with NAC are compared to equivalent data without NAC. (**B**) C81 cells were treated with Vehicle or different concentrations of Peptide A-8R, as shown, and 0–5 mM NAC, as shown, and monitored for cell number after 0–6 days of incubation as measured by MTT assay. Data points represent averages of three independent experiments plus standard deviations. Asterisks indicate statistical significance (P<0.04). (**C**) C81 cells were treated with Vehicle or different concentrations of Peptide A-8R, as shown, and with or without 5 mM NAC, as shown, and monitored DNA damage after 1 hr incubation as measured by Comet assay.

Since AKT over-expression failed to rescue Peptide A-treated cells (see Fig. S4D), we looked for a different mechanism of cytotoxicity. Interestingly, recent data have shown that the most effective cancer drugs, including cisplatin and doxorubicin, induce tumor cell death by elevating levels of reactive oxygen species (ROS) [16], [17]. In view of the rapid cytotoxicity induced by Peptide A (data not shown), we examined the possibility that ROS was involved. Indeed, Peptide A-8R treatment resulted in significant ROS induction in LNCaP cells within 30 minutes (Fig. 4A), while the sGCα1-negative PC-3 cells do not respond (Fig. S5A). Importantly, the inactive peptide, C-8R, fails to induce ROS generation (Fig. S5B). As expected, ROS generation in LNCaP cells rose when Peptide A-8R concentration was increased from 10 to 25 µM, but, surprisingly, decreased with 50 µM (Fig. 4A). These results suggest that ROS generation may be responsible for the Peptide A-8R cytotoxicity at the lower concentrations of 10 and 25 µM, but not at 50 µM.

**Figure 5:**
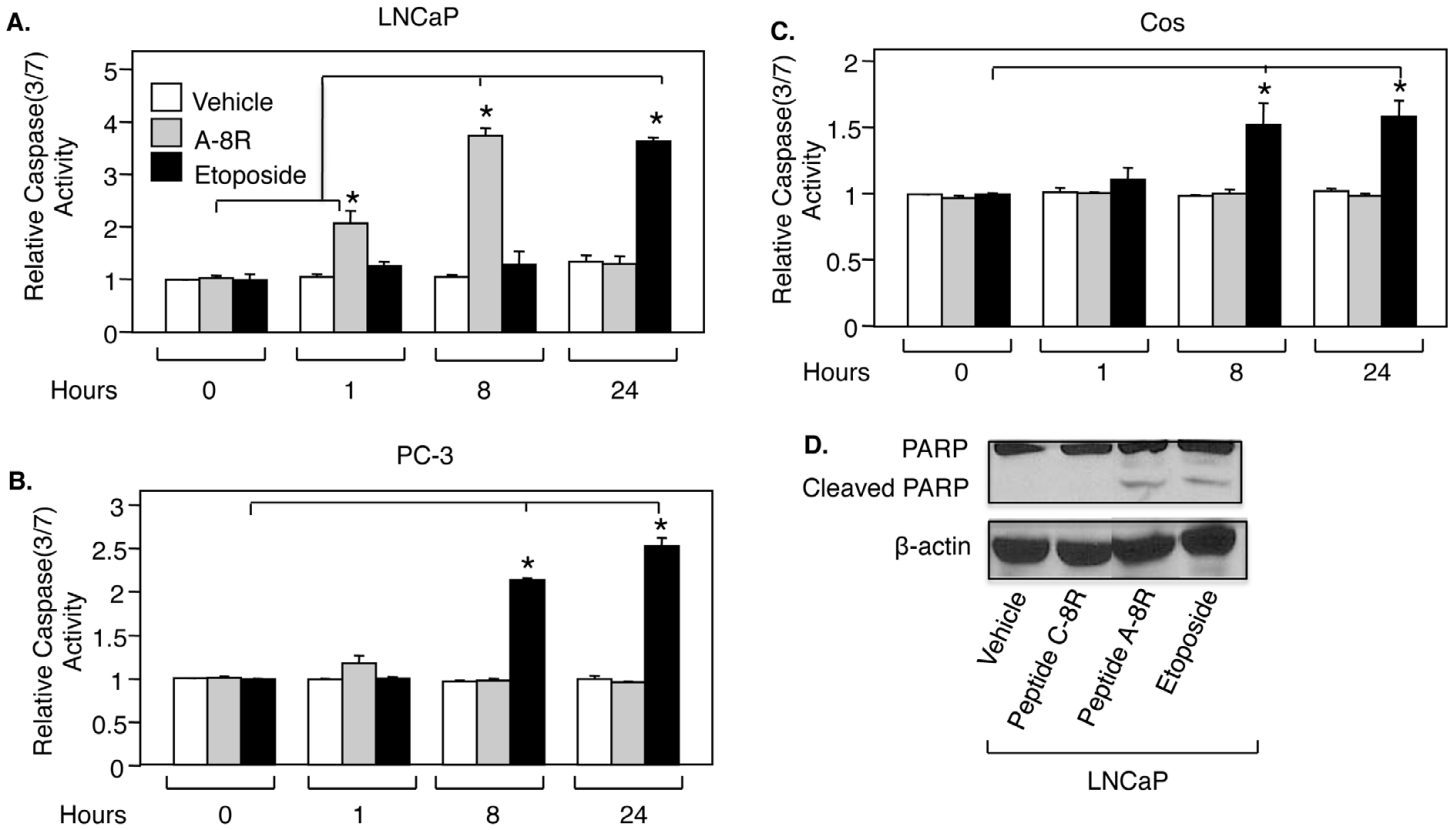
Peptide A-8R induces apoptosis of prostate cancer cells. (**A**) LNCaP, (**B**) PC-3, or (**C**) Cos cells were treated with Vehicle, Peptide A-8R (10 µM), or Etoposide (20 µM) for 0–24 hrs and subjected to a Caspase assay to measure apoptosis. Bar graphs represent averages of three independent experiments plus standard deviations. All activities are relative to the first condition, and this activity was set to 1. Asterisks indicate statistical significance (P<0.005). (**D**) LNCaP cells were treated with Vehicle, Peptide C-8R, Peptide A-8R, or Etoposide (each drug at 50 µM) for 8 hrs and monitored for apoptosis by measuring PARP cleavage using Western blotting. Note that β-actin was used to control for protein loading.

To address this possibility, we used the ROS scavenger NAC (N-acetyl cysteine) [18]. As shown in Fig. 5B, NAC was able to completely rescue the proliferation of cells treated with 10 µM Peptide A-8R or nearly completely with 25 µM. These NAC effects on proliferation correspond to the NAC effects on Peptide A-8R-mediated ROS generation (Fig. 4A), strongly suggesting that ROS generation is responsible for Peptide A cytotoxicity. Interestingly, NAC failed to significantly rescue the growth of cells treated with 50 µM Peptide A-8R (Fig. 4B). This finding, together with our earlier result showing minimal ROS induction with 50 µM Peptide A-8R (see Fig. 4A), argue that this high concentration of Peptide A-8R kills cells via mechanism independent of ROS generation.

ROS-induced cell death is associated with DNA damage [19], which is induced weakly by 10 µM Peptide A-8R and much more strongly by 25 µM, as measured by a Comet assay (Fig. 4C); importantly, this induction is completely blocked by NAC, clearly demonstrating the Peptide-A-8R-induced DNA damage requires ROS generation. Interestingly, 50 µM Peptide A-8R, which kills most of the cells, does not elicit a Comet signal (Fig. 4C). As shown in Fig. S6, this concentration of peptide kills the cells and disrupts their nuclei and DNA such that DAPI staining yields no signal and NAC had no effect.

### Peptide A Induces Apoptosis of Prostate Cancer Cells

Elevated levels of ROS can lead to apoptosis [20]. To confirm that the cytotoxicity of Peptide A-8R is through apoptosis, LNCaP cells were monitored for Caspase 3/7 activity. Peptide A-8R induced a 2-fold increase in Caspase activity after 1 hour of treatment, which increased to nearly 4-fold after 8 hrs (Fig. 5A), while the cytotoxic-deficient Peptide C-8R had no effect on Caspase activity (Fig. S5C). Caspase activity diminished to Vehicle levels after 24 hours of Peptide treatment, when most of the cells were dead. The positive control Etoposide induced similar levels of Caspase activity as did Peptide A-8R, but only after 24 hrs of treatment (Fig. 5A). Thus, Peptide A-8R was able to induce Caspase 3/7 activity much faster than the well-studied apoptosis-inducing drug Etoposide. To bolster these data, apoptosis was also monitored by measuring PARP cleavage, which was induced by Peptide A-8R to the same extent as by Etoposide (Fig. 5D). As expected, Peptide A-8R failed to induce apoptosis in PC-3 and Cos cells (Fig. 5B and 5C).

Interestingly, Peptide A-8R treatment also affects the expression of cell-cycle regulatory proteins. To begin this analysis, we measured in C81 cells the expression of p15 and p21, whose expression increased in response to Peptide A-8R but not Peptide C-8R, and of CDK6, which is unchanged in cells treated with Peptide A-8R (Fig. S7).

### Peptide A Inhibits the Growth of Castration-resistant Prostate Xenograft Tumors

**Figure 6:**
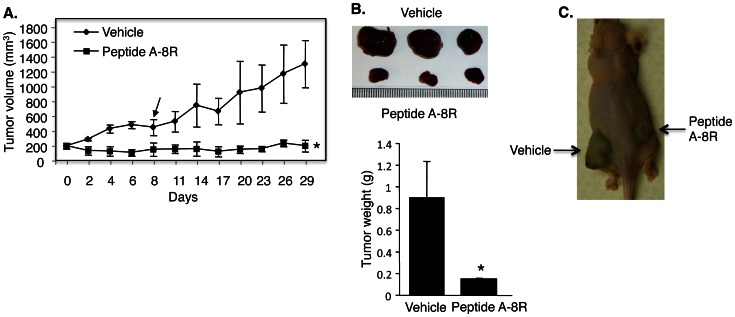
Peptide A-8R blocks the growth of castration-resistant prostate tumors. (**A**) Mouse xenograft tumors derived from C81 cells were treated with Vehicle or Peptide A-8R, after which they were allowed to grow for an additional three weeks without treatment. Data points represent average tumor size of three animals for each treatment plus standard deviations. Note that there is a statistical difference (P<0.00004) in average tumor size for each day of tumor measurement. Arrow represents day of last injection of Vehicle or Peptide A-8R. (**B**) Upper panel shows images of three Vehicle-treated and three Peptide A-8R-treated tumors that were excised from the animals and lower panel shows the average weight of these tumors. Bar graphs represent averages of each set of three tumors plus standard deviations. Asterisks indicate statistical significance (P<0.02). (**C**) Two tumors, one on each flank of the same animal, were treated with either Vehicle or Peptide A-8R.

The cytotoxic activity of Peptide A-8R on cultured prostate cancer cells suggests that it may have activity on tumorigenesis. In order to establish that Peptide A-8R can inhibit prostate tumors under physiological circumstances, it was necessary to show that the same effects observed in culture occur when tumors are grown in mice. This was initially analyzed using LNCaP cells to establish xenograft tumors in nude mice and direct injection of Peptide A-8R, which resulted in strong anti-tumor activity (Fig. S8). Since Peptide A-8R is strongly cytotoxic to hormone-refractory C81 cells, we next tested this Peptide’s activity in tumors derived from C81 cells. Peptide A-8R stopped tumor growth and actually caused some tumor regression during the five injections of peptide (Fig. 6A and 6B). Remarkably, the tumors did not grow at all three weeks after Peptide treatment was stopped, while the Vehicle-treated tumors grew by more than three-fold (Fig. 6A). To control for potential differences between animals, we tested Peptide A-8R and Vehicle in the same mouse having two tumors. As shown in Fig. 6C, the Peptide-treated tumor was markedly smaller than the Vehicle-treated tumor. In fact, the Peptide-treated tumor did not grow, as was observed in the other animals, while the Vehicle-treated tumor grew markedly. These data demonstrate a strong anti-tumor activity for Peptide A-8R in nude mice and suggest that this peptide may be effective against CRPC.

## Discussion

Current therapy used for prostate cancer targets either androgen production or AR activity [21]. Both of these therapies become ineffective when prostate cancer recurs in a castration-resistant state and the AR neither depends on androgens nor is antagonized by anti-androgens (i.e. Bicalutamide), and the outcome is usually death [21]. The ineffectiveness of these therapies against CRPC has led to recent developments of new antiandrogens, including MDV3100 [22], BMS-641988 [23], and ARN-509 [24]. However, even these new drugs have limited success against CRPC and some have significant side effects [24]–[26].

Our approach was to look downstream of AR, to an AR-regulated gene that is essential for the viability of cancer cells and progression of prostate cancer. sGCα1 is such a gene, promoting prostate cancer cell proliferation and survival [7] and mediating p53 inhibition [10]. This gene also exhibits an expression in prostate tumors that increases with advancing stages of cancer, exhibiting the highest expression levels in CRPC [7]. Taken together, these findings strongly suggest that sGCα1 is an attractive novel target; Peptide A-8R was designed to target this protein.

Since sGCα1 is expressed in both hormone-dependent and -independent AR-positive cells [7], our expectation was that Peptide A-8R should be cytotoxic to both types of prostate cancer cells. Indeed, our results demonstrate this to be the case, with two lines of hormone-independent cells, C81 [27] and CWR-22Rv1 [28] cells. Since these two cell lines are distinct and represent different tumors, their similar sensitivities to the cytotoxic activity of Peptide A-8R suggest that this peptide may have widespread activity against CRPC. Indeed, our mouse xenograft studies with C81 tumors demonstrate a strong anti-cancer activity for Peptide A-8R with intra-tumoral injection; intra-peritoneal injection did not work (data not shown) due to the absence of prostate cancer-targeting mechanism for Peptide A, an objective of future studies.

Because Peptide A-8R was targeted for sGCα1, it was important for us to demonstrate an interaction between these two molecules. This was done using multiple approaches. Immunocytochemistry showed that Peptide A-8R colocalizes with sGCα1 only in the cytoplasm of LNCaP cells, where endogenous sGCα1 is localized [10]. A physical association of Peptide A-8R with sGCα1 was demonstrated by Biotin pulldown, using a Biotin-labeled Peptide A-8R, which we showed has equal cytotoxicity to Peptide A-8R, verifying that addition of Biotin did not disrupt Peptide A-8R activity. However, neither assay allowed us to determine the binding affinity of Peptide A-8R for sGCα1, which we determined using a FITC-labeled Peptide A-8R. This binding affinity was determined to be about 12 µM, which falls well within the active range of Peptide A-8R cytotoxicity. While a higher binding affinity (in nM range) would be desirable, the µMolar affinity of Peptide A provides a clear advantage. This low affinity of the Peptide A-sGCα1 interaction, lower than the affinity of the sGCα1-sGCβ1 dimerization [29], makes it less likely that the Peptide will disrupt sGCα1 function in NO signaling [8]. This was confirmed by our data showing Peptide A-8R does not interfere with sGCα1-sGCβ1 dimerization and androgen-induced cGMP synthesis. Consistent with this, the Peptide had no effect on the behavior or weight of the mice in the animal studies, an important finding in view of the ubiquity of NO signaling in mammalian biology [8]. Our complementary study showed that Peptide A-8R cytotoxicity does not depend on NO signaling, as Peptide activity was not influenced by either chemical inhibition of sGC enzyme activity with ODQ or addition of 8-Br-cGMP or the NO sequestering agent SNP. Thus, Peptide A-8R has cytotoxic and anti-tumor activity without apparent dependence on or interference of NO signaling, and, therefore, likely is targeting the high levels of endogenous sGCα1 that is not associated with sGCβ1 [7], [10].

Because of the limited available knowledge of sGCα1 function in prostate cancer, it was challenging to determine the mechanism of cytotoxic action of Peptide A-8R. Our previous data showed that sGCα1 down-regulates p53 activity [10], making it possible that Peptide A attenuates this sGCα1 action and drives cells into p53-dependent apoptosis. Surprisingly, our data show that Peptide A-8R does not disrupt p53 activity nor depend on endogenous p53 for inducing apoptosis of prostate cancer cells (data not shown). sGCα1 has a second novel and important activity in prostate cancer cells, up-regulating the AKT protein. This was demonstrated in stable cell lines over-expressing sGCα1, which exhibited elevated levels of total AKT and phosphorylated AKT, resulting in increased levels of AKT targets, including GSK-3β and PDK1. Since PI3-AKT signaling has been shown to have pro-survival and pro-proliferative roles in prostate cancer [30], something we confirmed by using the PI3 inhibitor LY294002 [30], we hypothesized that Peptide A-8R will interfere with the sGCα1 positive effect on AKT. Indeed, our data demonstrate that Peptide A-8R significantly reduces AKT protein levels, in both prostate cancer cells and prostate tumors in mice. Surprisingly, however, viral over-expression of AKT failed to rescue Peptide A-treated cells. While we do not know what is responsible for this, Peptide A-8R may induce another change in prostate cancer cells, in addition to reduced AKT levels, that may be responsible for cytotoxicity. Thus, the reduced levels of AKT may result from cells dying through this other cytotoxic mechanism. This possibility of multiple modes of action of cancer drugs has been shown for several drugs, including Cisplatin and Doxorubicin [17]; our data here also suggest that Peptide A-8R may kill prostate cancer cells via two mechanisms, depending on concentration (10–25 vs 50 µM).

The cytotoxic mechanism for Peptide A-8R anti-cancer action is indeed a distinct mechanism. Our data clearly show that Peptide A-8R can induce generation of ROS and DNA damage in prostate cancer cells. While Peptide-treated cells were not rescued with AKT over-expression, they were efficiently rescued with NAC, a ROS sequestering agent [18]. Interestingly, there is a direct correlation between Peptide A-8R-induced cytotoxicity and ROS generation at 10 and 25 µM peptide concentration, but not at 50 µM. 50 µM Peptide A-8R has the strongest cytotoxic activity on cells but has the lowest induction of ROS, suggesting that a major part of cytotoxicity triggered by 50 µM Peptide A-8R is independent of ROS induction. This is supported by the NAC rescue experiment, in which cells treated with 50 µM Peptide A-8R were only marginally rescued by NAC, in contrast to the strong rescue observed at 10 and 25 µM. While the mechanism of cytotoxicity for 50 µM Peptide A is unknown, it is noteworthy that this concentration triggers a higher level of necrosis-dependent death than the lower concentrations (data not shown), implying that the higher concentration of Peptide A-8R depends on necrosis and lower concentrations depend on apoptosis. A similar observation has been made for several cancer cytotoxic drugs, including amphipathic fusion peptides against prostate cancer in which higher concentrations led to a significantly enhanced necrosis [31]. Future work can further evaluate this aspect of Peptide A-8R activity, as well as enhance its cytotoxic activity on and increase its targeting for prostate cancer cells. Its strong anti-tumor activity, ROS-mediated cytotoxicity, and novel target for prostate cancer all warrant further investigation of Peptide A-8R as a new and effective therapy for CRPC.

## Supporting Information

Figure S1 **Peptide A-8R does not affect sGC-NO signaling.** (**A**) Whole-cell extracts from LNCaP cells treated with Vehicle, Peptide A-8R, or C-8R were subjected to IP using an anti-sGCα1 or anti-sGCβ1 antibody and then probed by Western blotting for sGCα1 (Upper) or sGCβ1 (Lower). Input represents amount of sGCα1 or sGCβ1 found in extracts. (**B**) LNCaP cells grown in the absence or presence of 1 nM R1881 were treated with Vehicle, 20 µM Peptide A-8R, 20 µM C-8R, or 8-Br-cGMP and monitored for cGMP levels using the cGMP E1A kit (Enzo Life Sciences). (**C**) LNCaP cells grown in the absence or presence of 8-Br-cGMP were treated with Vehicle, Peptide A-8R, or ODQ and monitored for cell density using the MTT assay. (**D**) LNCaP cells were treated with Vehicle or different concentrations of Peptide A-8R and C-PTIO and monitored for cell density. Bar graphs represent averages of three independent experiments plus standard deviations. Asterisks indicate statistical significance (P<0.04).
(TIF)

Figure S2 **Peptide A-8R cytotoxicity depends on membrane translocation.** LNCaP cells were treated with Vehicle or different concentrations of Peptide A-8R or Peptide A and monitored for cell density using the MTT assay. Bar graphs represent averages of three independent experiments plus standard deviations. Asterisks indicate statistical significance (P<0.005).
(TIF)

Figure S3 **sGCα1-overexpressing LNCaP cells exhibit elevated levels of AKT.** (**A**) LNCaP cells were treated with Vehicle, LY290042, or U0126 and then monitored for cell density using the MTT assay. (**B**) Parental (LN) and two stable LNCaP cell lines (LNα1-4 and LNα1-6) over-expressing sGCα1 were monitored by Western blotting for expression of sGCα1, total AKT, S473-phosphorylated AKT, T308-phosphorylated AKT, phosphorylated PDK1, and phosphorylated GSK-3β. (**C**) Same cells were monitored for cell density using MTT assay. For A and C, bar graphs represent averages of three independent experiments plus standard deviations. (**D**) LNα1-6 cells were transfected with either control (−) or sGCα1 siRNA and monitored by Western blotting for expression of sGCα1, total AKT, S473-phosphorylated AKT, and T308-phosphorylated AKT. (**E**) LNCaP cells were transfected with either control or sGCα1 siRNA and monitored for cell density after 0–6 days of incubation. Data points represent averages of three independent experiments plus standard deviations. Asterisks indicate statistical significance (P<0.003). (**F**) Cells from above were monitored by Western blotting for expression of sGCα1. Note that β-actin was used to control for protein loading in B, D, and F. Asterisks indicate statistical significance (P<0.05).
(TIF)

Figure S4 **Peptide A-mediated down-regulation of AKT is not involved in its cytotoxicity to prostate cancer cells.** Western blotting was used to measure the levels of total AKT or Ser-473-phosphorylated AKT in (**A**) LNCaP cells treated with Vehicle, Peptide A-8R, or Peptide C-8R (25 µM) for different times, as shown, (**B**) mouse xenograft tumors treated with Vehicle or Peptide A-8R, or (**C**) LNCaP cells infected with Empty or AKT-expressing adenovirus and treated with Peptide A-8R at different concentration, as shown, for 30 min. Note that β-actin was used to control for protein loading. (**D**) LNCaP cells, infected with Empty or AKT-expressing adenovirus, were grown in 10% serum under different concentrations of Peptide A-8R, as shown. Cell number was measured after 0–6 days of incubation. Data points represent averages of three independent experiments plus standard deviations.
(TIF)

Figure S5 **ROS generation is not induced by Peptide A-8R in PC-3 cells and Peptide C-8R in LNCaP cells.** (**A**) PC-3 cells were treated with Vehicle or different concentrations of Peptide A-8R or Peptide C-8R, as shown, or (**B)** LNCaP cells were treated with Vehicle or different concentrations of Peptide A-8R or Peptide C-8R, or H2O2 and monitored for ROS generation after 0–30 (A) or 30 (B) min. (**C**) LNCaP cells were treated with Vehicle, Peptide C-8R (10 µM), Peptide A-8R (10 µM), or Etoposide (100 µM) for 0–8 hrs and subjected to a Caspase assay to measure apoptosis. Bar graphs represent averages of three independent experiments plus standard deviations. (C) All activities are relative to the first condition, and this activity was set to either 1. Asterisks indicate statistical significance (P<0.006) of all values compared to Vehicle (B) or to 0 hour (C).
(TIF)

Figure S6 **Cells treated with high concentration of Peptide A-8R failed to exhibit DAPI DNA staining.** C81 cells were first treated with or without 5 mM NAC for 2 hrs and then Vehicle or different concentrations of Peptide A-8R, as shown, for 1 hr and stained with DAPI. Phase contrast images are shown.
(TIF)

Figure S7 **Peptide A-8R induces expression of p15 and p21 in prostate cancer cells.** C81 cells treated with 25 µM Peptide A-8R or Peptide C-8R for different times, as shown, monitored for expression of p15, p21, and CDK6 by Western blotting (antibodies from Cell Signaling Technology). Note that β-actin was used to control for protein loading.
(TIF)

Figure S8 **Peptide A-8R blocks the growth of LNCaP prostate tumors.** Mouse xenograft tumors derived from LNCaP cells were treated with Vehicle or Peptide A-8R, after which they were allowed to grow for an additional three weeks without treatment. Data points represent average tumor size of three animals for each treatment plus standard deviations. Note that there is a statistical difference (P<0.0005) in average tumor size for each day of tumor measurement. Arrow represents day of last injection of Vehicle or Peptide A-8R.
(TIF)
