# Supplementary figures and images for: A Peptide against Soluble Guanylyl Cyclase α1: A New Approach to Treating Prostate Cancer
Source: PLoS One. 2013 May 27;8(5):e64189. doi: 10.1371/journal.pone.0064189 (PMC3664642; doi:10.1371/journal.pone.0064189)

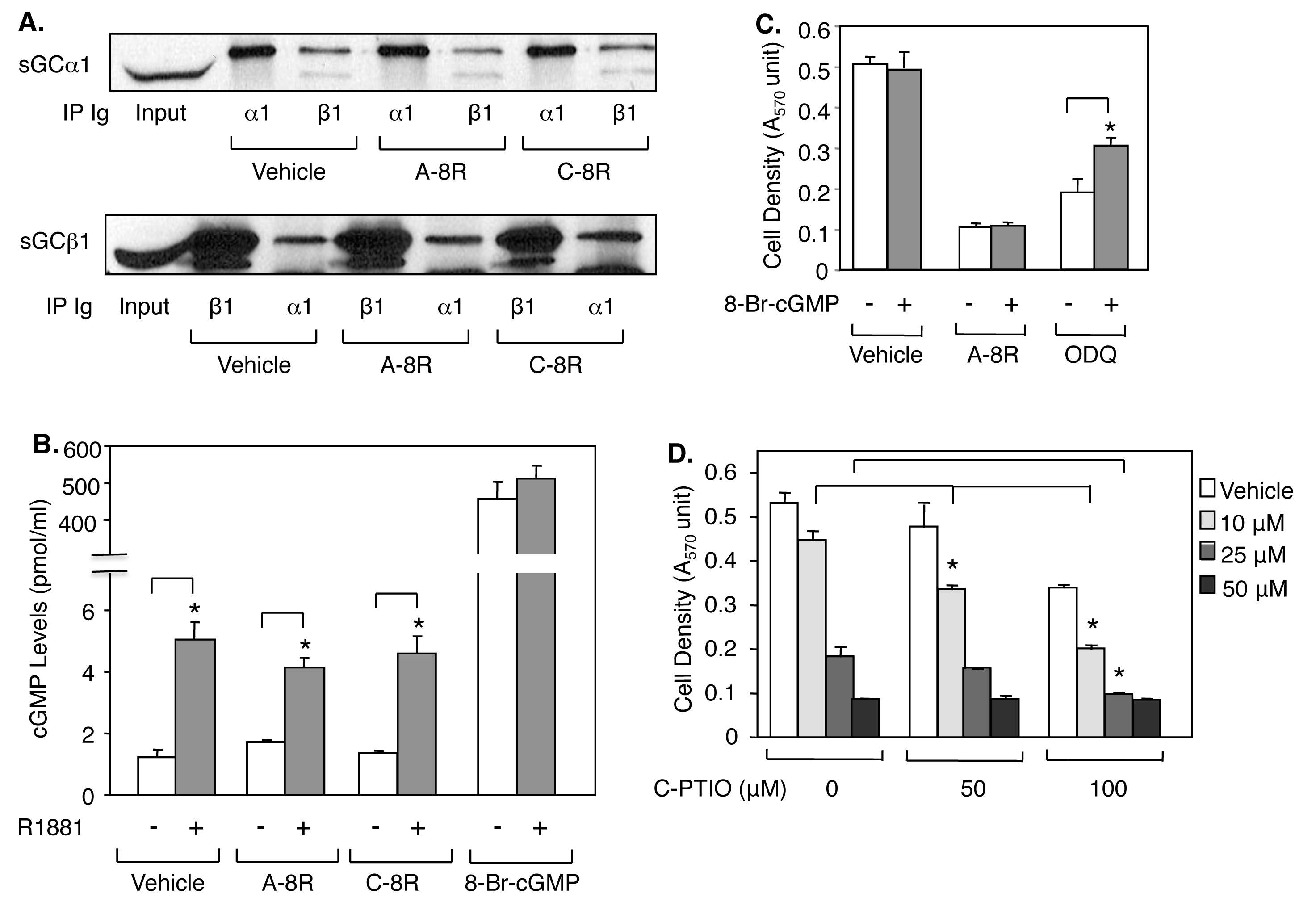

Supplement: Figure S1 — Peptide A-8R does not affect sGC-NO signaling. (A) Whole-cell extracts from LNCaP cells treated with Vehicle, Peptide A-8R, or C-8R were subjected to IP using an anti-sGCα1 or anti-sGCβ1 antibody and then probed by Western blotting for sGCα1 (Upper) or sGCβ1 (Lower). Input represents amount of sGCα1 or sGCβ1 found in extracts. (B) LNCaP cells grown in the absence or presence of 1 nM R1881 were treated with Vehicle, 20 µM Peptide A-8R, 20 µM C-8R, or 8-Br-cGMP and monitored for cGMP levels using the cGMP E1A kit (Enzo Life Sciences). (C) LNCaP cells grown in the absence or presence of 8-Br-cGMP were treated with Vehicle, Peptide A-8R, or ODQ and monitored for cell density using the MTT assay. (D) LNCaP cells were treated with Vehicle or different concentrations of Peptide A-8R and C-PTIO and monitored for cell density. Bar graphs represent averages of three independent experiments plus standard deviations. Asterisks indicate statistical significance (P<0.04). (TIF) [file pone.0064189.s001.tif]

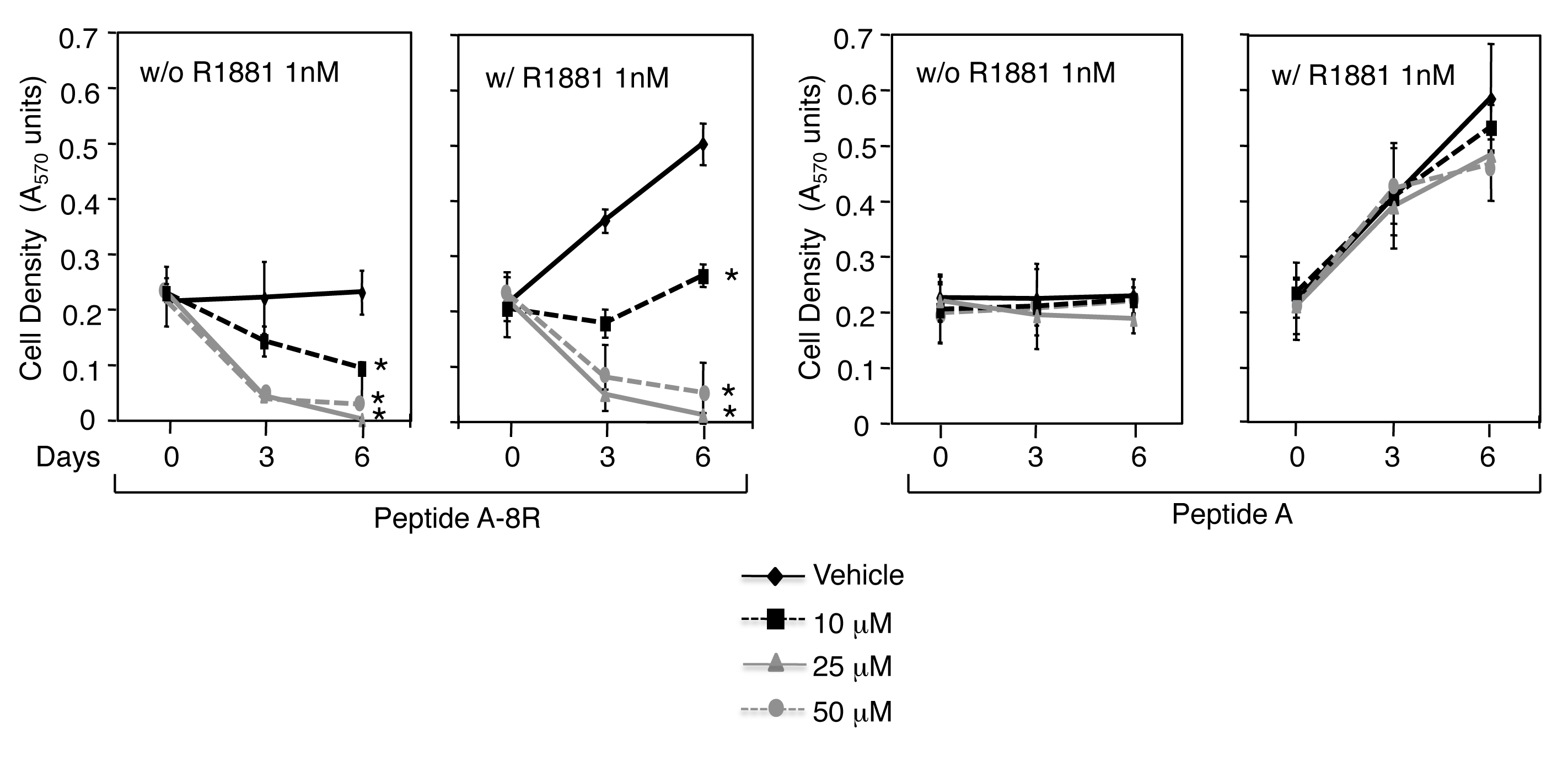

Supplement: Figure S2 — Peptide A-8R cytotoxicity depends on membrane translocation. LNCaP cells were treated with Vehicle or different concentrations of Peptide A-8R or Peptide A and monitored for cell density using the MTT assay. Bar graphs represent averages of three independent experiments plus standard deviations. Asterisks indicate statistical significance (P<0.005). (TIF) [file pone.0064189.s002.tif]

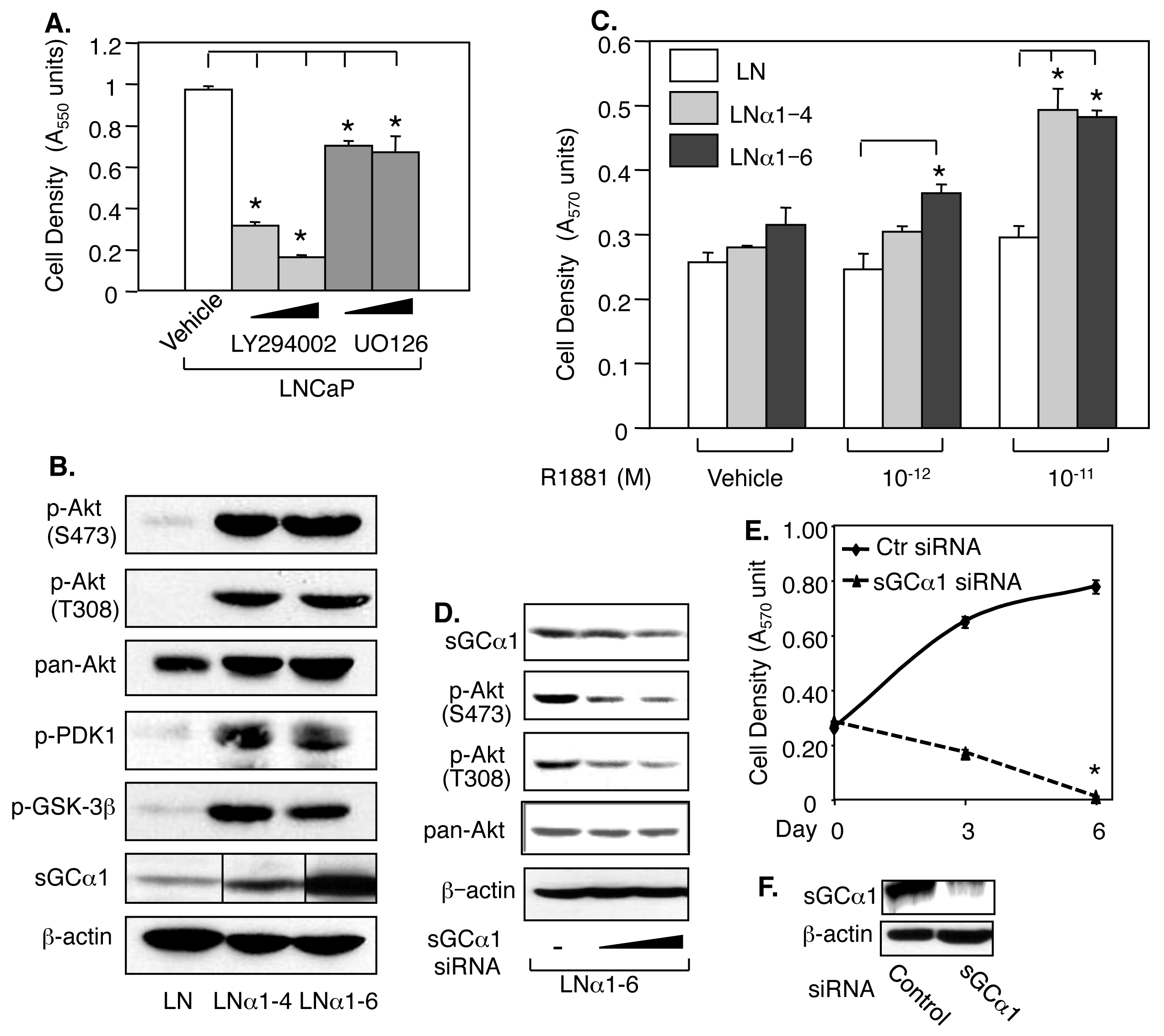

Supplement: Figure S3 — sGCα1-overexpressing LNCaP cells exhibit elevated levels of AKT. (A) LNCaP cells were treated with Vehicle, LY290042, or U0126 and then monitored for cell density using the MTT assay. (B) Parental (LN) and two stable LNCaP cell lines (LNα1-4 and LNα1-6) over-expressing sGCα1 were monitored by Western blotting for expression of sGCα1, total AKT, S473-phosphorylated AKT, T308-phosphorylated AKT, phosphorylated PDK1, and phosphorylated GSK-3β. (C) Same cells were monitored for cell density using MTT assay. For A and C, bar graphs represent averages of three independent experiments plus standard deviations. (D) LNα1-6 cells were transfected with either control (−) or sGCα1 siRNA and monitored by Western blotting for expression of sGCα1, total AKT, S473-phosphorylated AKT, and T308-phosphorylated AKT. (E) LNCaP cells were transfected with either control or sGCα1 siRNA and monitored for cell density after 0–6 days of incubation. Data points represent averages of three independent experiments plus standard deviations. Asterisks indicate statistical significance (P<0.003). (F) Cells from above were monitored by Western blotting for expression of sGCα1. Note that β-actin was used to control for protein loading in B, D, and F. Asterisks indicate statistical significance (P<0.05). (TIF) [file pone.0064189.s003.tif]

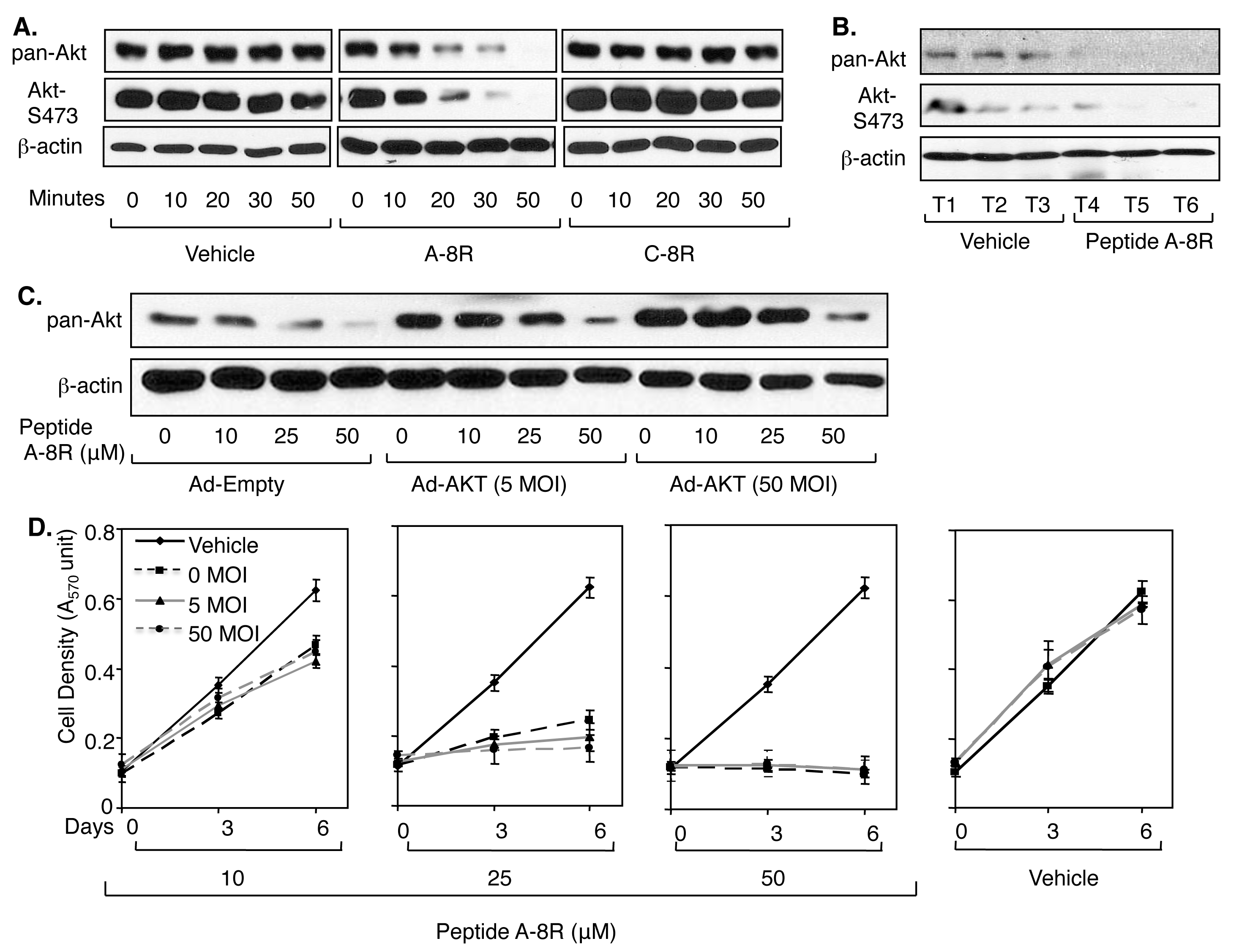

Supplement: Figure S4 — Peptide A-mediated down-regulation of AKT is not involved in its cytotoxicity to prostate cancer cells. Western blotting was used to measure the levels of total AKT or Ser-473-phosphorylated AKT in (A) LNCaP cells treated with Vehicle, Peptide A-8R, or Peptide C-8R (25 µM) for different times, as shown, (B) mouse xenograft tumors treated with Vehicle or Peptide A-8R, or (C) LNCaP cells infected with Empty or AKT-expressing adenovirus and treated with Peptide A-8R at different concentration, as shown, for 30 min. Note that β-actin was used to control for protein loading. (D) LNCaP cells, infected with Empty or AKT-expressing adenovirus, were grown in 10% serum under different concentrations of Peptide A-8R, as shown. Cell number was measured after 0–6 days of incubation. Data points represent averages of three independent experiments plus standard deviations. (TIF) [file pone.0064189.s004.tif]

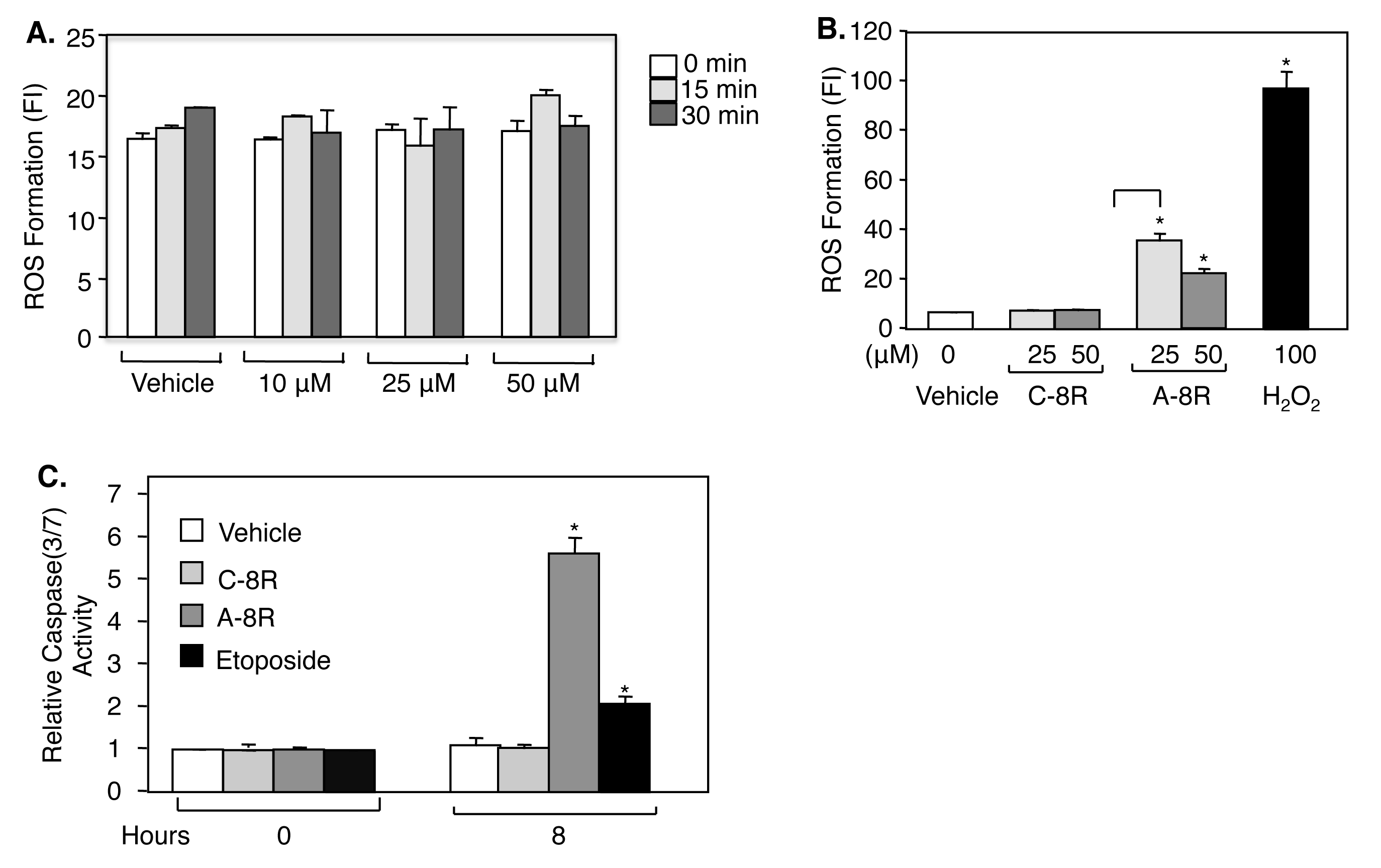

Supplement: Figure S5 — ROS generation is not induced by Peptide A-8R in PC-3 cells and Peptide C-8R in LNCaP cells. (A) PC-3 cells were treated with Vehicle or different concentrations of Peptide A-8R or Peptide C-8R, as shown, or (B) LNCaP cells were treated with Vehicle or different concentrations of Peptide A-8R or Peptide C-8R, or H2O2 and monitored for ROS generation after 0–30 (A) or 30 (B) min. (C) LNCaP cells were treated with Vehicle, Peptide C-8R (10 µM), Peptide A-8R (10 µM), or Etoposide (100 µM) for 0–8 hrs and subjected to a Caspase assay to measure apoptosis. Bar graphs represent averages of three independent experiments plus standard deviations. (C) All activities are relative to the first condition, and this activity was set to either 1. Asterisks indicate statistical significance (P<0.006) of all values compared to Vehicle (B) or to 0 hour (C). (TIF) [file pone.0064189.s005.tif]

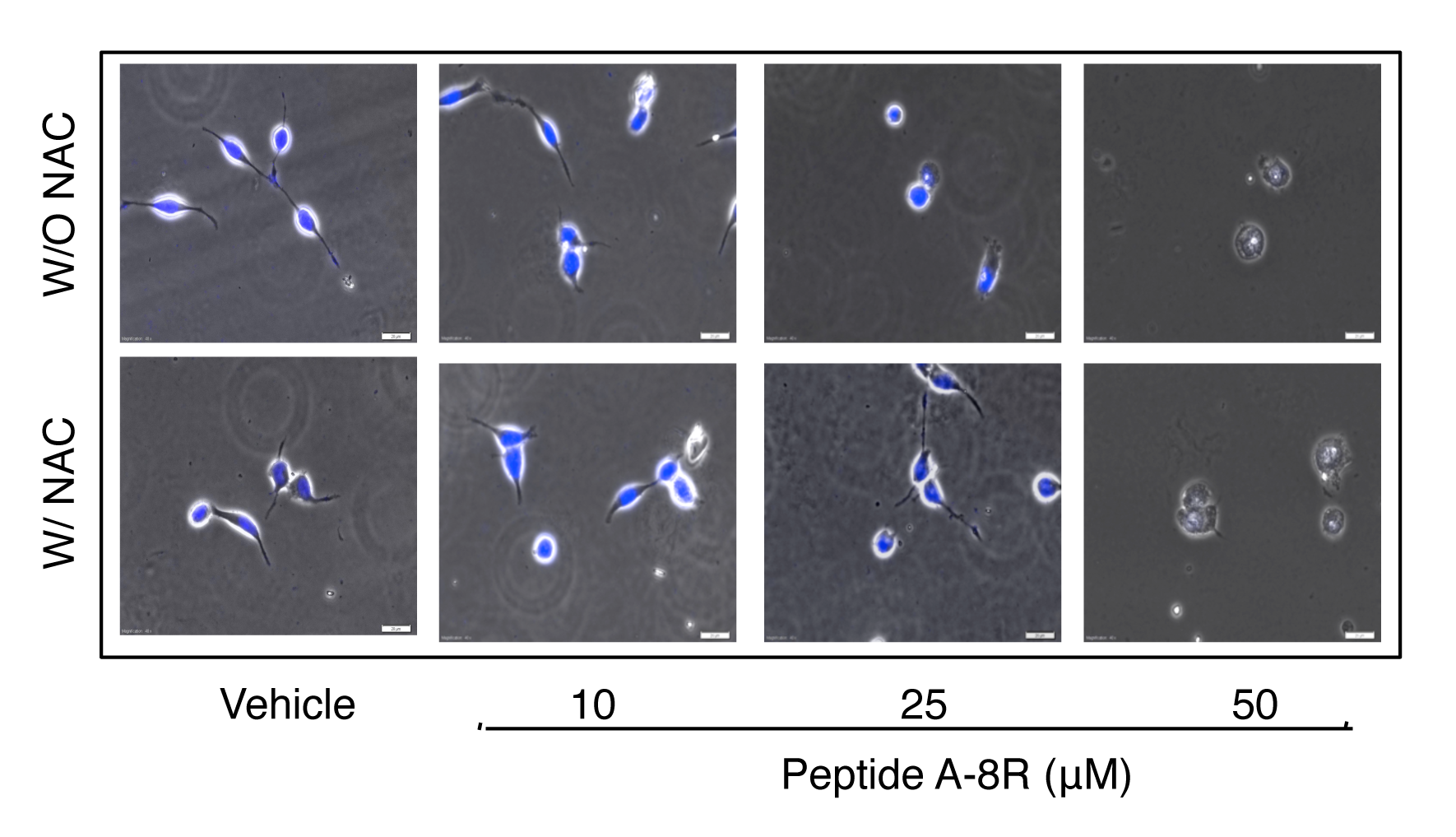

Supplement: Figure S6 — Cells treated with high concentration of Peptide A-8R failed to exhibit DAPI DNA staining. C81 cells were first treated with or without 5 mM NAC for 2 hrs and then Vehicle or different concentrations of Peptide A-8R, as shown, for 1 hr and stained with DAPI. Phase contrast images are shown. (TIF) [file pone.0064189.s006.tif]

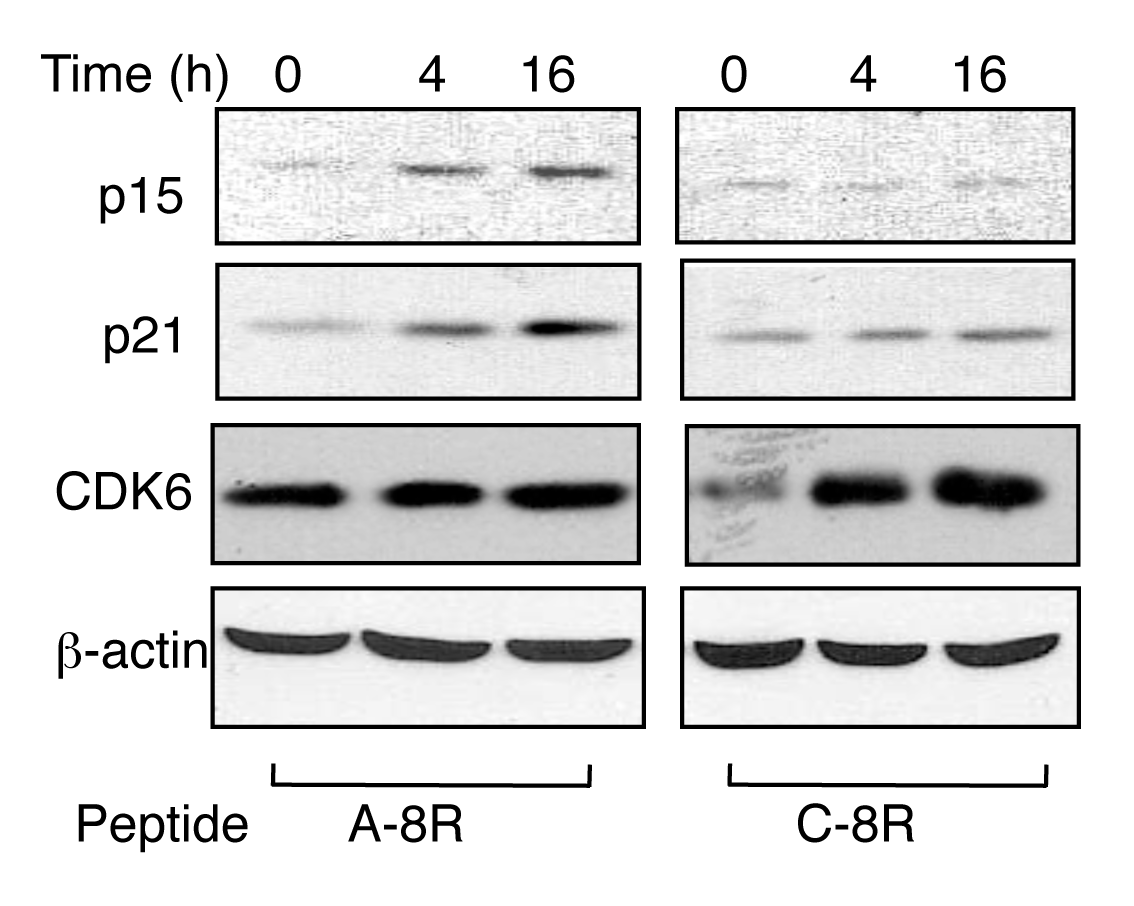

Supplement: Figure S7 — Peptide A-8R induces expression of p15 and p21 in prostate cancer cells. C81 cells treated with 25 µM Peptide A-8R or Peptide C-8R for different times, as shown, monitored for expression of p15, p21, and CDK6 by Western blotting (antibodies from Cell Signaling Technology). Note that β-actin was used to control for protein loading. (TIF) [file pone.0064189.s007.tif]

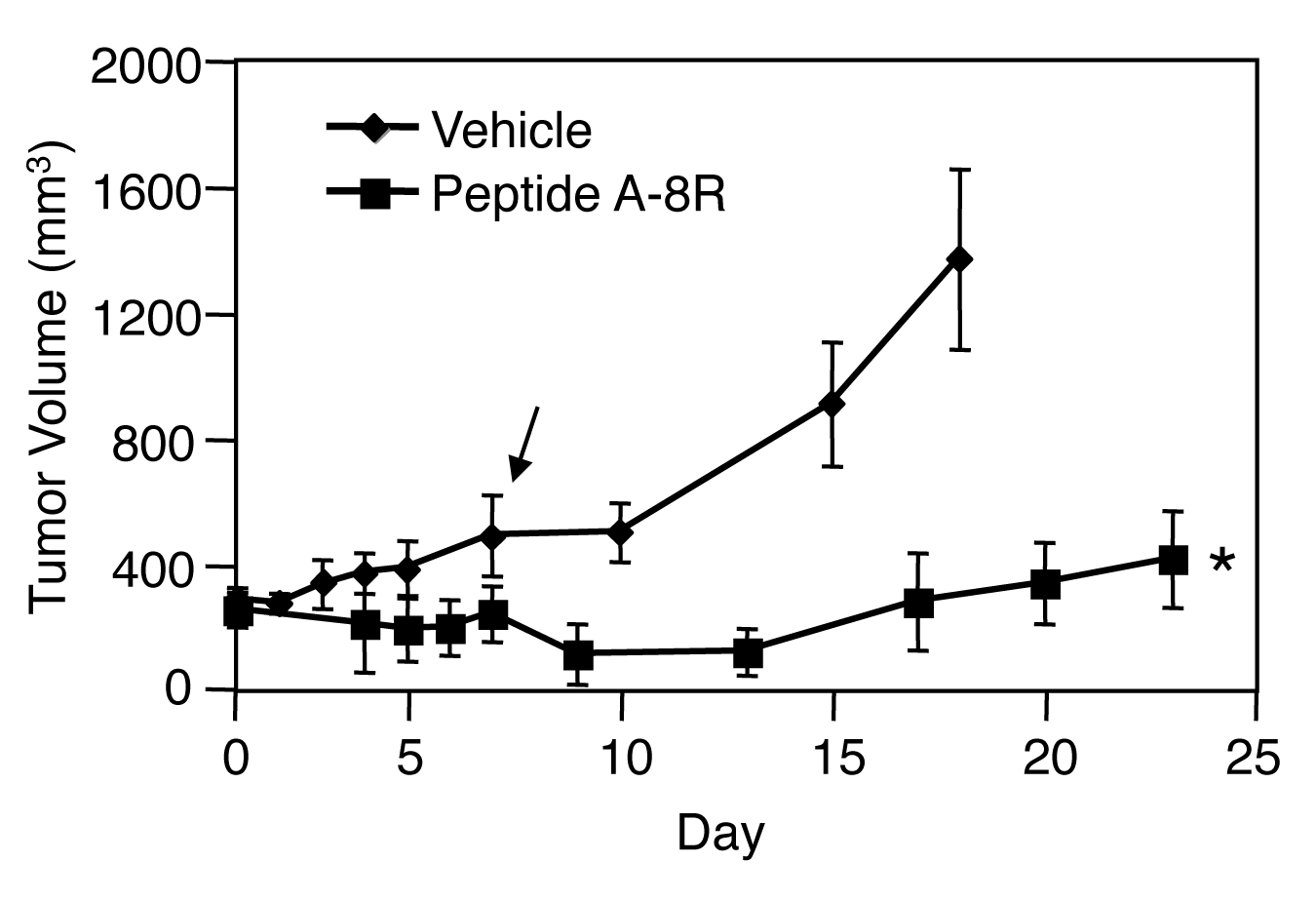

Supplement: Figure S8 — Peptide A-8R blocks the growth of LNCaP prostate tumors. Mouse xenograft tumors derived from LNCaP cells were treated with Vehicle or Peptide A-8R, after which they were allowed to grow for an additional three weeks without treatment. Data points represent average tumor size of three animals for each treatment plus standard deviations. Note that there is a statistical difference (P<0.0005) in average tumor size for each day of tumor measurement. Arrow represents day of last injection of Vehicle or Peptide A-8R. (TIF) [file pone.0064189.s008.tif]
